# Reactivation of *Mycobacterium simiae* after the recovery of COVID-19 infection

**DOI:** 10.1016/j.jctube.2021.100257

**Published:** 2021-06-25

**Authors:** Morteza Masoumi, Fatemeh Sakhaee, Farzam Vaziri, Seyed Davar Siadat, Abolfazl Fateh

**Affiliations:** a Department of Mycobacteriology and Pulmonary Research, Pasteur Institute of Iran, Tehran, Iran; b Microbiology Research Center (MRC), Pasteur Institute of Iran, Tehran, Iran

**Keywords:** COVID-19, *Mycobacterium simiae*, SARS-CoV-2

## Abstract

There are limited studies on the coinfection of coronavirus disease 2019 (COVID-19) with nontuberculous mycobacteria. Here, we briefly describe the reactivation of *Mycobacterium simiae* infection in a patient who had recovered from COVID-19 in October 2020, Iran. During the pandemic of COVID-19, other infectious agents should not be ignored.

## 1 Introduction

*Mycobacterium simiae* pulmonary disease could be easily confused with *M. tuberculosis* and most of *M. simiae* isolates are resistant to rifampin, isoniazid, and ethambutol [1]. Coronavirus disease 2019 (COVID-19) is known as a new viral infection that is caused by severe acute respiratory syndrome coronavirus 2 (SARS-CoV-2). Bacterial coinfection and reactivation of bacterial infection in patients with COVID-19 are poorly understood [2].

## 2 Case

The patient was a 52-year-old woman who complained of a pulmonary infection caused by *M. simiae* infection that lasted for the past two years. After treatment with clarithromycin, ethambutol, and ciprofloxacin, the patient successfully was recovered, and the symptoms of pulmonary infection were disappeared, and the results of the computed tomographic (CT) scan were normal.

For two days, she had close contact with a COVID-19 patient, and the symptoms, such as fever, dry cough, headache, tiredness, diarrhea, and loss of taste or smell, appeared after one week. The oxygen saturation was 96% in ambient air, and the COVID-19 real-time PCR test using nasopharyngeal swab was positive with normal CT scan. The patient was sent home and treated conservatively. After 20 days, all her symptoms were improved, and the result of real-time PCR for COVID-19 was negative.

After 25 days of COVID-19 recovery, she complained of pulmonary infection. The physician assumed the recurrence of COVID-19 infection, but the real-time PCR was negative for COVID-19 infection. The CT scan indicated bronchiectasis, consolidation, nodular lesions, and infiltration in the left lung with a degree of atelectatic changes. All clinical parameters were normal, except erythrocyte sedimentation rate (ESR) (82 mm/h) and C-reactive protein (CRP) rate (85.1 mg/L). Her symptoms were fever, sputum, night sweats, productive cough, weight loss, and dyspnea**.**

The three sputum samples were sent to the Pasteur Institute of Iran in October 2020 to evaluate the presence of mycobacterial infection. The smear test indicated acid-fast bacilli (AFB) in all three sputum samples. The results of culture after five weeks revealed slowly-growing mycobacteria with smooth*,* small, and non-pigmented colonies, when exposed to light, turned into yellow colonies.

The multilocus sequence analysis was also performed using full *16S rRNA* gene and partial *rpoB/hsp65* genes, as described previously [3], [4]. The results of gene sequencing showed 100% homology to *M. simiae*.

The susceptibility testing was performed according to the Clinical & Laboratory Standards Institute (CLSI) guidelines for clarithromycin, amikacin, ethambutol, moxifloxacin, ciprofloxacin, trimethoprim/sulfamethoxazole (TMP/SMX), streptomycin, isoniazid, ethambutol, levofloxacin, rifampin, rifabutin, and linezolid [5]. The results indicated that the isolate was extremely susceptible to amikacin, levofloxacin, and clarithromycin. Based on the susceptibility data, the patient was treating of levofloxacin, clarithromycin, and amikacin. After three month of treatment, the rate of CRP and ESR were normal and the general condition of the patient is very good and the symptoms of the disease almost improved.

## 3 Discussion

To the best of our knowledge, this is an important report of reactivation of prior *M. simiae* infection following COVID-19. There are several studies on co-infection with tuberculosis (TB) and COVID-19 and also reactivation of latent-TB to acute TB after COVID-19 infection [6], [7]. However, limited data are available on the COVID-19 infection and nontuberculous mycobacteria infection. In a study, the COVID-19 coinfection with *M. abscessus* was reported in a patient with multiple myeloma [8].

In this study, however, the normal CT scan results during the COVID-19 infection and appearing the symptoms at her second presentation suggested onset of acute *M. simiae* infection was acute.

Several reports have indicated that CD4+/CD8+ T-cells play a vital role in defence against mycobacterial infection [6], [9]. In COVID-19 patients have been shown a significant depletion in the count of T-cell lymphocytes [10]. The count of both CD4+ and CD8+ were severely decreased, and surviving T-cells appear functionally exhausted. This depletion and dysfunction of T-cell may increase the development of latent TB to active TB [6]. It seems that a similar mechanism may have occurred in our patient.

## 4 Conclusion

Even when patients seem to be primarily infected with COVID-19 and are severely ill, determination of other superimposed infections is necessary, and finally, proper antimicrobial therapy can change the outcomes.

## Declaration of Competing Interest

The authors declare that they have no known competing financial interests or personal relationships that could have appeared to influence the work reported in this paper.
